# Rampant Cheating by Pathogens?

**DOI:** 10.1371/journal.ppat.1005792

**Published:** 2016-09-08

**Authors:** Ethan A. Rundell, Saria A. McKeithen-Mead, Barbara I. Kazmierczak

**Affiliations:** 1 Department of Microbial Pathogenesis, Yale University, New Haven, Connecticut, United States of America; 2 Department of Medicine (Infectious Diseases), Yale University, New Haven, Connecticut, United States of America; The University of North Carolina at Chapel Hill, UNITED STATES

Pathogenic bacteria often cooperate during infection. Communication through quorum sensing allows bacteria to coordinate gene expression in a population density–dependent manner; biofilm formation increases population resistance to host- and antibiotic-related stresses. Although such cooperative behaviors benefit pathogens at the population level, individual cells experience costs of cooperation. “Cheater” mutants may arise that avoid these costs while exploiting the “public goods” produced by cooperative cells.

Recent research has highlighted that, surprisingly, the bacterial type III secretion system (T3SS) can serve as a “public good” during infection. This structure is produced by multiple Gram-negative bacteria and is used to inject proteins that modulate host biology (effectors) directly into host cells. Often, T3SS-secreted effectors alter immune responses in a way that benefits bacteria at a local population level in the infected host. This cooperative function is subject to exploitation by cheater mutants lacking a T3SS but benefiting from secretion by wild-type cooperators. Here, we review examples of T3SS cheaters, consider fitness costs associated with producing the T3SS, and discuss mechanisms that can reduce the risk of cheater emergence in infected hosts.

## Immunosuppression as a Public Good: *Pseudomonas aeruginosa* and *Yersinia pestis*

A common function of T3SS effectors is suppression of the host immune response. Two recent papers provide examples of T3SS-mediated immunosuppression as a public good subject to exploitation by T3SS-negative cheaters.

In a mouse model of infection by *Pseudomonas aeruginosa*, mutants lacking a T3SS are defective in establishing infections on their own but thrive when coinfected with isogenic wild-type *P*. *aeruginosa* or *P*. *aeruginosa* constitutively expressing the T3SS [1]. In these coinfections, the T3SS-negative mutants (cheaters) initially outcompete T3SS-positive cells but subsequently lose their fitness advantage over time. Cheating does not occur in immunodeficient or immunosuppressed mice and is dependent on the *P*. *aeruginosa* T3SS effector ExoU, a phospholipase A_2_ that leads to host phagocytic cell necrosis. These results suggest that ExoU-mediated immunosuppression in mice is a public good subject to exploitation by cheaters [1]. An avirulent laboratory *Escherichia coli* strain is able to grow in the mouse lung when coinfected with *P*. *aeruginosa* in an ExoU-dependent manner, providing further evidence that ExoU acts as a public good through immunosuppression [2].

Similarly, the *Yersinia pestis* T3SS contributes to evasion of host immunity within infected animal models [3]. Mutants lacking the plasmid encoding the T3SS are defective in establishing mouse lung infections on their own but are able to survive, grow to high numbers, and spread beyond the initial site of infection when coinfected with T3SS-positive bacteria [3,4]. These findings suggest that in *Y*. *pestis*, the T3SS also acts as a public good through immunosuppression. Supporting this model, multiple other normally avirulent *Y*. *pestis* mutants grow in vivo when coinfected with wild-type *Y*. *pestis* [4].

## Inflammation and Invasion as Public Goods: *Salmonella typhimurium*

*Salmonella enterica* subspecies 1 serovar typhimurium (*S*. *typhimurium*) uses its T3SS to induce, rather than suppress, inflammation and compete with the host microbiota [5]. *S*. *typhimurium* mutants lacking a T3SS are defective in establishing mouse infections on their own but outcompete isogenic wild-type bacteria in coinfections, indicating that the *S*. *typhimurium* T3SS functions as a public good [6]. When these secretion-negative cheater mutants outcompete wild-type *S*. *typhimurium*, the population becomes defective in establishing new mouse infections [7].

*S*. *typhimurium* invades host epithelial cells in a T3SS-dependent manner. Cooperative invasion of epithelial cells has been observed in vitro: mutants lacking a T3SS are defective in cell invasion but improve in invasion in the presence of wild-type bacteria [8]. However, *S*. *typhimurium* cheaters in mouse gut infections remain predominantly in the gut lumen and are underrepresented in the gut epithelium, suggesting that the ability of cheaters to invade the epithelium in vivo is highly limited [7].

## Why Cheat? Costs of Secretion

Public goods come with a cost, and avoiding this cost permits cheaters to thrive. What is the cost of the T3SS? *S*. *typhimurium* T3SS-negative mutants grow more quickly than wild-type *S*. *typhimurium* in vitro, suggesting a general fitness cost of producing the T3SS [6]. This is not the case in *P*. *aeruginosa*, yet cheating in vivo still occurs for this pathogen [1]. Additional selection pressures against maintaining a T3SS in vivo may involve the host immune system, as seen for *Citrobacter rodentium*, whose locus of enterocyte effacement (LEE) encodes a T3SS [9]. The mouse adaptive immune system preferentially targets and clears *C*. *rodentium* that produce the LEE virulence factors—rather than mutants lacking the LEE genes—in an antibody-dependent manner [9]. The T3SS also activates innate immune signaling [10], raising the possibility that cheaters may benefit in vivo by avoiding activation of the innate immune system as well as avoiding clearance by the adaptive immune system; however, this remains to be tested.

## How Do Microbial Populations Prevent the Rise of Cheaters?

When cheaters lacking a T3SS dominate a bacterial population, virulence, transmission to new hosts, and in vivo fitness for the population are compromised [1,6,7]. Several possible mechanisms reduce the risk of T3SS-negative cheater emergence, but there is also evidence that in some cases, cheaters still emerge and outcompete cooperators.

### Private Goods

Unlike public goods, private goods benefit only the cells producing them. When public and private goods are coregulated, cheaters cannot produce private goods and thus experience a direct cost of cheating. For example, a gene required for adenosine metabolism in *P*. *aeruginosa* is controlled by the LasR/I quorum sensing system, and cheaters lacking this quorum sensing system experience fitness defects when adenosine is the primary carbon source [11].

Do similar dynamics exist for the T3SS? In *P*. *aeruginosa*, only the effector ExoU serves as a public good. The effectors ExoS and ExoT, which inhibit phagocytosis, function as private goods in infected mouse lungs, likely because T3SS-negative cheaters eventually encounter phagocytic cells not intoxicated by T3SS-expressers [1]. In *S*. *typhimurium*, T3SS-expressing bacteria invade the gut epithelium, while cheaters, which have an advantage in the lumen, have limited ability to invade [7]. The ability to colonize new niches favors persistence of T3SS-expressing bacteria and serves as a private good. In contrast, *Y*. *pestis* cheaters lacking a T3SS can spread beyond the initial site of infection [4]. Bacterial pathogens produce effectors of diverse function [reviewed in 12,13], suggesting that similar dynamics may occur for other bacteria, although this has not been demonstrated experimentally.

### Population Bistability

Many of the experimental systems cited use mixtures of genetically fixed T3SS-positive and T3SS-negative bacteria to demonstrate that cheating can occur in vivo. In many cases, isogenic populations of these pathogens show heterogeneity in their secretion phenotypes under identical growth conditions. In *S*. *typhimurium*, this heterogeneity reduces the emergence of genetically avirulent cheaters. Individual cells phenotypically negative for secretion avoid the associated fitness costs and successfully compete with avirulent cheaters that spontaneously arise while maintaining the genetic information needed to produce public goods. In a *S*. *typhimurium* strain engineered to have a smaller, phenotypically negative subpopulation, avirulent cheaters emerged more rapidly, inflammation ceased prematurely, and bacteria were cleared from the gut. These findings nicely demonstrate how phenotypic heterogeneity reduces the threat of cheating and stabilizes “cooperative virulence” [6].

### Cheaters Never Win?

Despite mechanisms to reduce the risk of cheater emergence, cheaters may still arise in infected hosts. In the insect pathogen *Bacillus thuringiensis*, cheaters lacking the public good toxin Cry are retained long-term in infected hosts [14]. In chronic human infections with *P*. *aeruginosa*, cooperative behaviors such as quorum sensing are often lost over time in clinical isolates. Of note, T3SS-negative strains have been isolated from individuals with both acute and chronic *P*. *aeruginosa* infections, raising the intriguing possibility that cheating occurs during human infections [15–18].

## Future Directions

Cheating for the T3SS has been demonstrated in a limited number of bacterial pathogens to date. Interestingly, although the T3SS is a public good in *Y*. *pestis*, it does not appear to serve as a public good in the closely related *Y*. *pseudotuberculosis*, suggesting species-specific variation in the T3SS as a public good [4]. Future work should clarify the extent to which the T3SS serves as a public good in other bacterial pathogens.

Spatial variation within infected hosts plays a major role in pathogenesis: for instance, *P*. *aeruginosa* clinical isolates from cystic fibrosis patients evolve independently within spatially isolated regions of the lung [19]. The spatial scale over which the T3SS can act as a public good in an infected host remains an open question and one that may vary by pathogen. Additionally, in pathogens where the T3SS is expressed heterogeneously, interactions between expression status and location in the host are largely unexplored. Although the *Y*. *pseudotuberculosis* T3SS does not appear to be a public good, expression of its T3SS within isogenic populations is associated with proximity to host cells, as assessed by fluorescent reporters [20]. Future studies should address whether this is also true in vivo for other pathogens as well as the consequences of this spatial variation in phenotype for bacteria and host.

Continued basic research focusing on the function, regulation, structure, and fitness costs of the T3SS in vitro and in vivo will be critical for understanding cooperative aspects of bacterial pathogenesis.
